# Longitudinal Examination of Everyday Executive Functioning in Children With ASD: Relations With Social, Emotional, and Behavioral Functioning Over Time

**DOI:** 10.3389/fpsyg.2018.01774

**Published:** 2018-10-10

**Authors:** Vanessa M. Vogan, Rachel C. Leung, Kristina Safar, Rhonda Martinussen, Mary Lou Smith, Margot J. Taylor

**Affiliations:** ^1^Diagnostic Imaging, The Hospital for Sick Children, Toronto, ON, Canada; ^2^Department of Applied Psychology and Human Development, Ontario Institute for Studies in Education, Toronto, ON, Canada; ^3^Department of Psychology, University of Toronto, Toronto, ON, Canada; ^4^Institute of Child Study, Ontario Institute for Studies in Education, University of Toronto, Toronto, ON, Canada

**Keywords:** Autism Spectrum Disorder, executive functioning, longitudinal, psychopathology, social functioning, anxiety, depression, aggressiveness

## Abstract

Executive functioning (EF) deficits are well-documented in Autism Spectrum Disorder (ASD), yet little is known about the longitudinal trajectory of “everyday” EF and links to social, emotional and behavioral outcomes in ASD. This study examined the profile of everyday EF utilizing parent-reported measures over 2 years, and explored whether prior estimates of EF were related to later co-morbid psychopathology and social functioning in 39 children with ASD and 34 typically developing (TD) children (ages 7–14 years). According to parent reports, children with ASD had impaired scores of EF in all domains at both time points, and showed no significant improvement across 2 years, compared to controls. Regression analyses showed that prior estimates of behavior regulation difficulties at time 1 uniquely predicted later emotional (i.e., symptoms of anxiety/depression) and behavioral (i.e., oppositionality/aggressiveness) problems in children with ASD 2 years later. Furthermore, an improvement of metacognitive skills predicted a reduction of social difficulties over 2 years in ASD. These results imply that EF may be a potential target of intervention for preventing and reducing co-morbid psychopathology and promoting social competence in youth with ASD. Furthermore, the findings that EF related to behavior is more critical for later emotional and behavioral functioning, whereas EF related to *cognition* is more critical for social functioning, indicates that it may be beneficial to tailor treatment. Future studies investigating the effectiveness of EF-based interventions in improving the cognitive, psychological and social outcomes in ASD are of high priority.

## Introduction

In addition to social-communicative deficits and repetitive/restricted behaviors and interests, individuals with Autism Spectrum Disorder (ASD) often have executive functioning (EF) impairments (Hill, 2004; Russo et al., 2007; Kenworthy et al., 2008; Bramham et al., 2009; Gardiner et al., 2017). EF is higher-order cognitive processes that regulate goal-directed behavior by enabling individuals to disengage from the immediate context for the coordination and execution of future goals. EF difficulties are now explicitly described as diagnostic features of ASD in the Diagnostic and Statistical Manual of Mental Disorders-Fifth Edition (DSM-5; American Psychiatric Association [APA], 2013). Research has demonstrated that individuals with ASD have particular difficulty in aspects of planning and cognitive/behavioral flexibility (Gioia et al., 2002; Hill, 2004; Kenworthy et al., 2005; Rosenthal et al., 2013), but also in task initiation (Bramham et al., 2009), working memory (Andersen et al., 2015b), self-monitoring (Russell, 2002), and inhibition (Lemon et al., 2011). Impairments of EF in ASD are correlated with symptom presentation (Lopez et al., 2005; Hill and Bird, 2006; Kenworthy et al., 2009; Reed et al., 2013), adaptive behavior problems (Gilotty et al., 2002; Pugliese et al., 2016), social competence (Pellicano, 2010; Leung et al., 2016; Torske et al., 2016), academic success and psychiatric co-morbidities (Lawson et al., 2015), and are associated with greater dependence and poor outcomes in adulthood (Hume et al., 2009). EF depends on prefrontal cortices which have a protracted maturation (Powell and Voeller, 2004; Sowell et al., 2004), making EF susceptible to developmental disturbances. Thus, one crucial question is how the EF profile manifests over time in ASD and its impact on developmental outcomes.

It is well-documented that EF develops throughout childhood and into adulthood in normative populations (Best and Miller, 2010). Cross-sectional and longitudinal studies of individuals with ASD generally support age-related maturation of EF through childhood and adolescence, but they are developmentally delayed (Happé et al., 2006; Chen et al., 2016) and often remain impaired compared to same-age typical peers (Luna et al., 2007; see O’Hearn et al., 2008 for a review; Pellicano, 2010; Andersen et al., 2015a), or show no improvement of working memory over time (Andersen et al., 2015b). These studies rely on laboratory based assessments of EF, which are performance measures. Although the highly controlled conditions created by lab-based tasks allow for the assessment of optimal EF performance, they do not accurately represent the complexity of children’s daily lives and may be less sensitive for measuring EF deficits in ASD. Thus, some question the ecological validity and generalizability of these measures (Gioia et al., 2002; Kenworthy et al., 2008; Blijd-Hoogewys et al., 2014), particularly because EF problems in everyday life (e.g., informant-reported) are observed in individuals with ASD even when lab-based measures of EF are intact (Kenworthy et al., 2008). Unlike lab tasks, everyday observations of EF take place in a social context under real-world expectations, in which children with ASD may be more susceptible than typically developing (TD) children. Thus, gathering information from parents about EF in everyday situations that are less structured is critical. The handful of studies that have examined the cross-sectional development of everyday EF in ASD report no improvement of EF with age or even age-related declines (Rosenthal et al., 2013; van den Bergh et al., 2014), whereas typical populations show developmental improvements (Huizinga and Smidts, 2010). This discrepancy may be due to the fact that children with ASD are more vulnerable than TD children to the increasing complexity of environmental demands as they progress into adolescence, which is often controlled in standardized lab measures of EF (van den Bergh et al., 2014). The above studies are cross-sectional, and thus, longitudinal studies are warranted to advance our understanding of the developmental trajectories of EF in everyday settings, and whether these skills have an impact on other developmental outcomes in ASD.

We were also interested in examining social, emotional and behavioral functioning as a means to measure the capacity for individuals with ASD to achieve optimal outcomes. Profound social deficits are central to the disorder, involving social pragmatic impairments, poor speech prosody, limited understanding of linguistic conventions, difficulties expressing emotions, and problems with interpersonal interactions and theory of mind (American Psychiatric Association [APA], 2013). In the emotional/behavioral functioning domain, ASD is associated with higher rates of co-morbid symptoms of both internalizing (e.g., depression and anxiety) and externalizing (e.g., aggressiveness and oppositionality) problems than the population at large (Bauminger et al., 2010; Rosenberg et al., 2011; Strang et al., 2012). Anxiety and behavioral disorders are among the most common psychiatric comorbidities in ASD (Simonoff et al., 2008). However, literature shows that there are more internalizing problems (withdrawal, social problems, anxiety and depression) than externalizing problems in ASD (Sturm et al., 2004). Given that social and emotional/behavioral problems are highly prevalent in children with ASD and there may be diagnostic challenges in identifying these conditions based solely on mental health assessment, it is crucial to identity risk factors and early predictors associated with the development of such symptoms.

Impairment in EF is one factor of importance for the development of social and emotional problems in ASD. Previous studies of TD children and young adults have found strong associations between poor EF and externalizing and internalizing behaviors (Castaneda et al., 2008; Hughes and Ensor, 2008; Snyder, 2013). Furthermore, longitudinal studies of TD children show that early EF difficulties *predict* later internalizing and externalizing problem behavior (Riggs et al., 2003; Martel et al., 2007), and social competence (Spinrad et al., 2006), suggesting that EF is critical for developmental processes. In a review article, Hofmann et al. (2012) also outline that working memory, inhibition and cognitive flexibility may generally subserve successful self-regulation. Prior research examining the relation between EF and social, emotional and behavioral functioning in individuals with ASD is limited. Some of these studies report concurrent relations between poor EF and high levels of anxiety, depression and aggression in youth with ASD, controlling for IQ (Hollocks et al., 2014), age and sex (Lawson et al., 2015). A study of adults with ASD found that EF components were differentially associated with certain emotional disorders, with cognitive flexibility associated with anxiety and planning/organization associated with depression (Wallace et al., 2016), above and beyond attentional problems. In contrast, other studies have failed to find both concurrent and longitudinal relations between laboratory tests of EF and severe mood dysregulation problems (Simonoff et al., 2012), behavioral difficulties, and emotional symptoms (Andersen et al., 2015a,b) in children with ASD. With regards to social deficits in ASD, studies have reported links between social difficulties and weaknesses of various EF processes, including task initiation, working memory and cognitive flexibility (Gilotty et al., 2002; Leung et al., 2016). Furthermore, Pellicano (2010) reported developmental links between early lab-based measures of EF and autistic children’s emerging theory of mind skills.

Another cognitive skill important for developing cognitive abilities and executive functions, is metacognition, the knowledge and monitoring of one’s own cognitive processes (Flavell, 1979). Metacognition emerges in childhood when children understand how they can learn, for instance (Paulus et al., 2014; Sobel and Letourneau, 2015), but continues to develop through adolescence (Weill et al., 2013). Metacognitive training is effective in improving children’s EF skills (Cornoldi et al., 2015), including those with ASD (Grainger et al., 2016; Maras et al., 2017). Difficulties in metacognitive functions are frequently reported in ASD and can impact a number of areas of competency (Grainger et al., 2014; Brosnan et al., 2016; McMahon et al., 2016), including social functioning (Torske et al., 2016).

In summary, these findings provide compelling evidence that one source of variability in social, emotional and behavioral outcomes in ASD are individual differences in the development of EF. However, surprisingly little is known about the predictive linkages between early *every day* EF skills and later social and psychological outcomes in ASD. The present study extends prior work by characterizing the *longitudinal* changes of EF, as well as metacognition, as observed in everyday settings, and investigates whether prior estimates of EF difficulties predict later social functioning and psychopathology (i.e., anxious/depressive symptoms and oppositionality/aggressiveness) in youth with ASD across 2 years. As abilities on laboratory tasks may differ from real-world observations, the current study focused on parent-reported EF measures using the Behavior Rating Inventory of Executive Functioning (BRIEF; Gioia et al., 2000), which captures daily scenarios of EF in individuals with ASD. We examined two EF domains, behavioral regulation and metacognition, to elucidate the emergence of these related but distinguishable skills (Miyake et al., 2000), as distinct cognitive processes may be differentially associated with outcomes. Overall, knowledge of developmental trajectories of everyday EF in ASD offers insight into the cognitive profile of the disorder, which is extremely informative for parents, educators and clinicians. Furthermore, an enhanced understanding of EF and metacognitive development in ASD and their links to future social and emotional functioning may allow for better assessment and will inform treatment planning for targeting cognitive, psychological and social outcomes.

## Materials and Methods

### Participants

We utilized data from a longitudinal study of brain and behavior neuroimaging in children with and without ASD (2011–2013). The complete sample for this study consisted of 73 participants: 39 children with ASD (34 males) and 34 age-matched TD (20 males). At baseline, children were between 7 and 14 years old (TD *M* = 11.2 years, *SD* = 2.1; ASD *M* = 10.6 years, *SD* = 1.8), and were followed up at approximately 2 years later, when they were between the ages of 9 and 16 years old (TD *M* = 13.3 years, *SD* = 2.1; ASD *M* = 12.9 years, *SD* = 1.8). Clinical diagnosis of ASD was confirmed in all of the children in the ASD group with a combination of expert clinical judgment, clinical records and the Autism Diagnostic Observation Schedule (ADOS; Lord et al., 2000) or the Autism Diagnostic Observation Schedule, Second Edition (ADOS-2; Lord et al., 2012), which was administered by a trained assessor who maintains inter-rater research reliability. All ASD participants had the primary diagnosis of ASD and although children with ASD often show signs of anxiety or ADHD, none were included who had other primary diagnosed psychiatric comorbidities, overt neurological damage or prematurity. Diagnosed developmental delay, learning disability and attention deficit hyperactivity disorder (ADHD) were used to exclude control children. All participants possessed a Full-Scale IQ estimate at 80 or above measured by the Wechsler Abbreviated Scale of Intelligence (WASI; Wechsler, 1999) – two subtest version. Full Scale IQ estimates were taken from the participants’ first evaluation (i.e., at baseline). **Table 1** provides information on the characteristics of the sample at both time points.

**Table 1:** Participant characteristics, including the *T*-scores for the everyday assessments.

	TD (*N* = 34)		ASD (*N* = 39)		Difference test
	%	Mean (*SD*)	%	Mean (*SD*)	
Sex (male)	59		87		χ^2^ = 7.6, *p* < 0.01
Age at T_1_ (years)		11.2 (2.1)		10.6 (1.9)	n.s.
Age at T_2_ (years)		13.3 (2.1)		12.9 (1.8)	n.s.
IQ		115.4 (11.7)		103.3 (14.7)	*t*_(71)_ = 3.9, *p* < 0.01
ADOS Total Score		n/a		12 (3.43)	
BRIEF (BRI) T_1_		45.32 (8.46)		69.77 (12.56)	See results section for analyses of these factors
BRIEF (BRI) T_2_		44.50 (8.30)		67.46 (14.32)	
BRIEF (MCI) T_1_		45.94 (9.01)		67.21 (9.33)	
BRIEF (MCI) T_2_		44.59 (7.65)		65.90 (10.82)	
SRS T_1_		45.06 (7.79)		79.14 (13.47)	
SRS T_2_		44.56 (5.65)		72.08 (12.15)	
CBCL anxious/depressed		52.62 (4.95)		67.26 (12.04)	
CBCL aggressiveness		51.74 (3.93)		59.77 (9.08)	

*N.B. The scores for the BRIEF, SRS and CBCL are T-scores. Higher scores indicate more behavioral and emotional problems; T-scores ≥ 65 represent clinical impairment.*

### Procedure

Children were recruited through community support centers, parent support groups, email lists, hospital ads, and private schools. Inclusion criteria were assessed through pre-screening interviews. All children provided informed assent, and the parents provided informed written consent. Clinical and cognitive testing and parent questionnaires were completed at baseline (T_1_) and approximately 2 years later (T_2_) at the Hospital for Sick Children in Toronto, Ontario. Intelligence and clinical testing batteries were consistent across time points. The parent questionnaires provided were the same at both time points, with an additional questionnaire measuring emotional and behavioral functioning at T_2_. This study was approved by the Hospital for Sick Children Research Ethics Board.

### Measures

#### Executive Functioning

The Behavior Rating Inventory of Executive Functioning, Parent Form (BRIEF; Gioia et al., 2000) was completed by parents at both time points. The BRIEF is an 86-item informant report questionnaire that assesses EF in everyday settings during the past 6 months (i.e., real world EF) for children and adolescents between 5 and 18 years old. The BRIEF has six subscales that are collapsed into two main indices: the Behavior Regulation Index (BRI), which includes three scales (Inhibit, Emotional Control and Shift), and the Metacognition Index (MCI), which includes five scales (Initiate, Organize/Plan, Organization of Materials, Working Memory, and Monitor). The present study utilized T scores (see **Table 1**). Higher scores are indicative of more EF problems, with T scores ≥ 65 representing clinical symptomatology. The BRIEF has acceptable reliability and well-established internal consistency, and convergent, discriminant, content validity (Gioia et al., 2000).

#### Emotional and Behavioral Functioning

The Child Behavior Checklist (CBCL; Achenbach and Rescorla, 2001) was completed by parents at T_2_. The CBCL is an informant report questionnaire that evaluates behavior and emotional symptoms during the past 6 months for children between 5 and 18 years old. The questionnaire is made up of 113 items that yield eight syndrome scales, six DSM-IV oriented scales, and three broader band scales. The Anxious/Depressed and Aggressive Behavior syndrome scales were of interest in the current study. The Anxious/Depressed scale assesses symptoms of both anxiety and depression, while the Aggressive Behavior scale consists of symptoms consistent with oppositionality, conduct and disruptive behaviors. The present study utilized *T*-scores (**Table 1**). Higher scores are indicative of more behavioral and emotional problems, with *T*-scores ≥ 65 representing clinical impairment. The CBCL has demonstrated good psychometric properties overall (Ivanova et al., 2007), as well as in identifying psychopathology in ASD (Gjevik et al., 2015).

#### Social Functioning

The Social Responsiveness Scale (SRS; Constantino and Gruber, 2005) was completed by parents at both time points. The SRS is a 65-item informant report that measures the range of severity of social impairment in ASD across the entire range of the spectrum, from non-existent to severe. Items assess social awareness, social cognition, social communication, social motivation, restricted interests and repetitive behavior. SRS Total *T*-scores were used for the purpose of the current study (summarized in **Table 1**). Higher SRS Total scores are indicative of greater social impairment, and *T*-scores ≥ 65 represent clinical symptomatology.

### Data Analysis

All statistical analyses were performed with SPSS for Macintosh, Version 24.

#### Longitudinal Trajectory of Everyday Executive Functions

Mixed between-within ANOVAs were conducted to assess the interaction between group (ASD and TD) and time on everyday EF. Significant Group × Time interactions were followed up with repeated measures ANOVAs for each group. Separate analyses controlling for the effect of age at baseline were conducted using an ANCOVA.

#### Relations Between EF and Emotional/Behavioral Symptomatology

Pearson correlations were completed to examine the bivariate relations among predictor variables (BRIEF BRI and MCI at T_1_) and outcome variables (CBCL Anxious/Depression and Aggressive Behavior at T_2_) within each group. EF variables and potential covariates that correlated significantly (*p* < 0.05) with CBCL scales were included as predictors in subsequent regression analyses. To examine whether prior estimates of EF at T_1_ predicted emotional/behavioral functioning at T_2_, a series of simple univariate regressions for each group were conducted using T_2_ CBCL scale scores (Anxious/Depressed and Aggressiveness) as dependent variables. Measures of EF were analyzed separately using simple univariate regressions due to strong correlations between the two predictor variables of interest (BRIEF MCI and BRI; TD: *r* = 0.68, *p* < 0.001; ASD: *r* = 0.59, *p* < 0.001), and a series of partial correlations were completed to understand the unique contribution of MCI and BRI to T_2_ CBCL variables. Due to the number of comparisons in these models, we used Bonferroni correction. IQ and sex were not correlated with study variables and thus were not entered into regression models.

#### Relations Between EF and Social Functioning

As measures of EF and social functioning were collected at *both* time points, we had the benefit of exploring the relationship between *change of EF* and *change of social functioning* over time (T_2_ –T_1_). Change scores were computed by subtracting *T*-scores at T_1_ from T_2_; negative change scores indicate improvement of abilities because higher *T*-scores reflect *more* problems. First, in order to describe the developmental trajectory of social functioning over time, mixed ANOVAs were conducted with Group as the between factor and Time as the within factor. A separate analysis controlling for the effect of age at baseline was conducted using an ANCOVA.

Bivariate Pearson correlation analyses between potential co-variates (IQ and sex), predictor variables (BRIEF BRI_T2-T1_ and MCI_T2-T1_), and outcome variables (SRS Total_T2-T1_) were completed. EF variables and potential covariates that correlated significantly (*p* < 0.05) with the outcome variable were included as predictors in subsequent regression analyses. To determine whether a change of EF predicted a change in social functioning from T_1_ to T_2_, simple univariate regressions were computed (see rationale above). IQ and sex were not correlated with study variables, and were excluded from the regression analysis. To note, the sample for this analysis consisted of 32 TD children and 37 children with ASD, after excluding participants who failed to complete the SRS at *both* time points; demographic data varied minimally, with no meaningful differences from the sample described in **Table 1**.

## Results

### The Development of Everyday EF Over 2 Years

**FIGURE 1:**
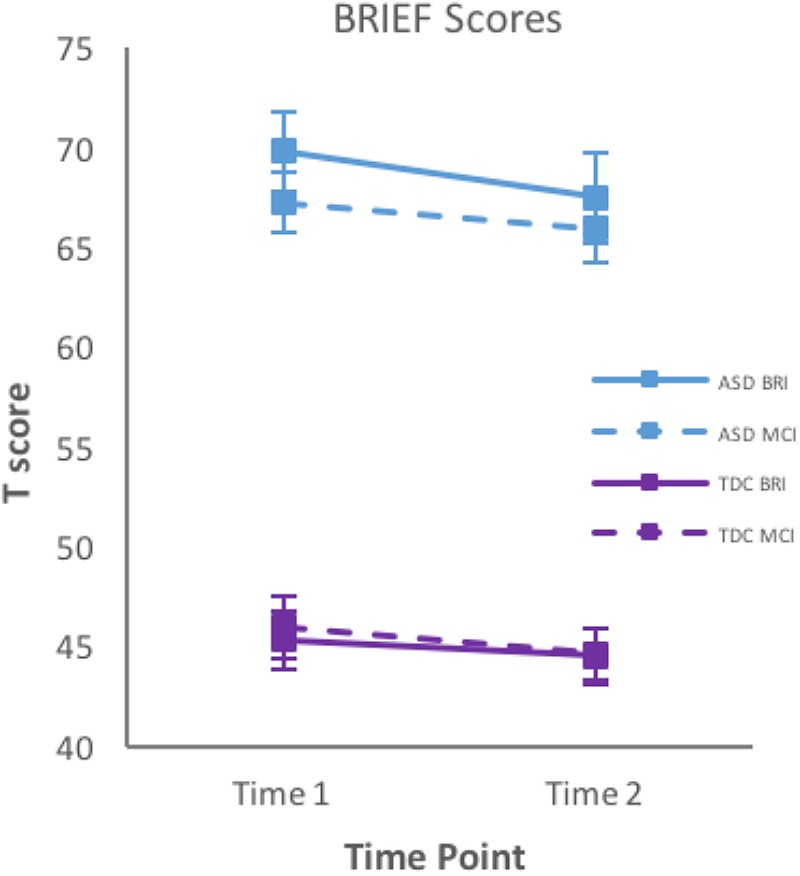
Mean T scores and standard error bars of parent-reported BRIEF Behavior Regulation Index (BRI) and Metacognition Index (MCI) at time 1 and time 2. BRIEF, Behavior Rating Inventory of Executive Functioning; ASD, Autism Spectrum Disorder; TD, typically developing children.

The longitudinal course of EF abilities for BRI and MCI is presented in **Figure 1**. For children’s BRIEF scores, mixed ANOVAs revealed no significant Group × Time interaction on BRI or MCI. When sex was included in the model, there were no effects or interactions, thus we collapsed across sex for further analyses. While there was no significant effect of time, a significant effect of group was found (*p* < 0.001), with participants in the ASD group showing more impaired scores on measures of BRI and MCI than control children. In terms of BRIEF individual subtest scores that make up the BRI, there was no significant Group × Time interaction on inhibition, shift and emotional control. However, there was a significant effect of Group (*p* < 0.001), with impaired scores on Inhibition, Shifting and Emotional Control for participants with ASD. Furthermore, there was a significant effect of time on Inhibition (*p* = 0.04). *Post hoc* testing using a repeated measures ANOVA for each group revealed a significant improvement of scores of Inhibition over time for children with ASD only (*p* = 0.02). In terms of BRIEF subtest scores that make up the MCI, there was no significant Group × Time interaction on any subtest scores (Initiation, Working Memory, Planning/Organization, Organization of Materials, Monitor). There was a significant group effect (*p* < 0.001), with the ASD group showing more impairments on all subscales, but there was no effect of time. The longitudinal course of individual BRIEF subscale scores is presented in **Supplementary Figure 1**. To examine whether initial age at baseline (T_1_) affected the change in EF over 2 years, we controlled for age and found no significant Time × Age interaction on any scales or indices of the BRIEF, indicating that effects of time were not impacted by initial age of the participants.

### Do Prior Estimates of EF Predict Later Emotional and Behavioral Functioning?

Independent sample *t-*tests were performed to determine whether children with and without ASD differed on emotional and behavioral symptoms at T_2._ Results indicate that children with ASD had significantly more parent-reported symptoms of anxiety/depression (ASD *M* = 67.26, *SD* = 12.04; TD *M* = 52.62, *SD* = 4.95; *t*_(52)_ = 6.95, *p* < 0.001) and aggressive/oppositional behavior (ASD *M* = 59.77, *SD* = 9.08; TD *M* = 51.73, *SD* = 3.93; *t*_(53)_ = 5.02, *p* < 0.001) than TD children (degrees of freedom adjusted for Levene’s tested for equality of variances).

Preliminary Pearson’s correlations showed that BRI problems at T_1_ were associated with greater symptoms of anxiety/depression (*r* = 0.45, *p* < 0.01) and aggressiveness (*r* = 0.61, *p* < 0.001) 2 years later in children with ASD. Furthermore, more MCI problems at T_1_ in ASD were also significantly correlated with greater symptoms of aggressiveness 2 years later (*r* = 0.41, *p* = 0.01) (see **Table 2**). In contrast, prior estimates of EF were not correlated with any later emotional/behavioral symptoms in TD children, and thus, regressions were not computed for this group.

**Table 2:** Correlation matrixes of EF variables and emotional and behavioral functioning variables for (A) TD children and (B) children with ASD.

	1	2	3	4	5	6
**(A) TD Group**						
(1) CBCL anxious/Depressed T_2_	–					
(2) CBCL aggressiveness T_2_	**0.41^∗^**	–				
(3) BRIEF BRI T_1_	0.23	0.28	–			
(4) BRIEF MCI T_1_	-0.04	-0.05	**0.68^∗∗∗^**	–		
(5) IQ	-0.05	0.22	0.29	0.13	–	
(6) Sex	0.20	0.04	-0.06	-0.20	0.22	–
**(B) ASD Group**						
(1) CBCL anxious/Depressed T_2_	–					
(2) CBCL aggressiveness T_2_	**0.52^∗∗∗^**	–				
(3) BRIEF BRI T_1_	**0.45^∗∗^**	**0.61^∗∗∗^**	–			
(4) BRIEF MCI T_1_	0.30	**0.41^∗∗^**	**0.59^∗∗∗^**	–		
(5) IQ	-0.20	-0.25	-0.11	0.03	–	
(6) Sex	-0.04	0.06	-0.06	-0.04	-0.02	–

*^∗^p < 0.05, ^∗∗^p < 0.01, ^∗∗∗^p < 0.001. Significant values after correction for multiple comparisons are in bold.*

Regression analyses showed that in children with ASD, more BRI problems at T_1_ predicted later symptoms of anxiety/depression (*p* < 0.01) at T_2_, accounting for 18% of adjusted variance. Furthermore, BRI (*p* < 0.001) and MCI (*p* = 0.01) difficulties at T_1_ predicted later aggressive behavior, accounting for 36% and 14% of adjusted variance, respectively (see **Table 3**). The unstandardized regression coefficients of BRI and MCI in models predicting Aggressiveness were not significantly different [*t*_(64)_ = 0.26, *p* = 0.79]. However, the partial correlation between MCI_T1_ and CBCL Aggressiveness_T2_ was no longer significant when BRI was partialled out (*r* = 0.071, *p* = 0.67), indicating that the variance in aggressive behavior explained uniquely by MCI is very minimal in ASD. To note, BRI_T1_ was significantly correlated with later Aggressiveness even when controlling for MCI (*r* = 0.50, *p* = 0.001). All reported regression models survived Bonferroni correction for multiple comparisons.

**Table 3:** Simple univariate regression analyses in the ASD group: Influence of EF at T_1_ on emotional/behavioral and social functioning at T_2_ for children with ASD.

Variable	Adjusted *R*^2^	*F*	*B*	SE *B*	*t*	Partial correlation
Predicting CBCL anxious/Depressed at T_2_						N/A
BRI	0.18	9.18	0.43	0.14	**3.03^∗∗^**	
Predicting CBCL aggressiveness at T_2_						
BRI	0.36	22.07	0.44	0.09	**4.70^∗∗∗^**	*r* = 0.50^∗∗∗^
MCI	0.14	7.23	0.40	0.15	**2.71^∗∗^**	*r* = 0.07
Predicting change in SRS total from T_1_ to T_2_						N/A
MCI Change	0.18	8.72	0.43	0.14	**2.95^∗∗^**	

*^∗^p < 0.05, ^∗∗^p < 0.01, ^∗∗∗^p < 0.001. Bolded values, significant after correction for multiple comparisons using Bonferroni.*

### Do Changes in EF Predict a Change in Social Functioning Over Time?

**FIGURE 2:**
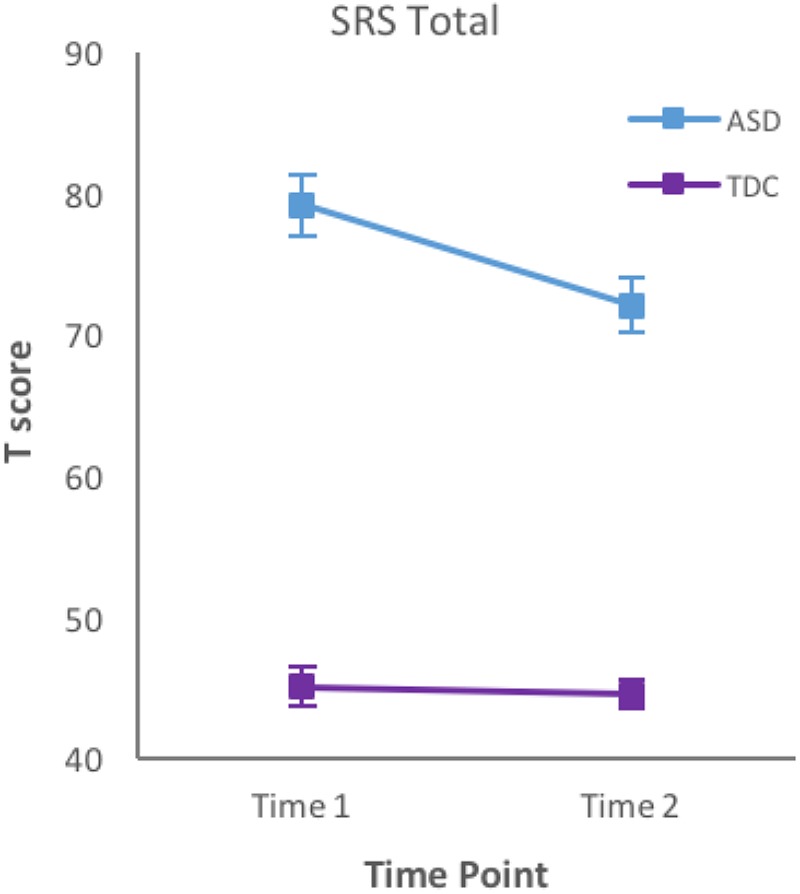
Mean T scores and standard error bars of parent-reported SRS Total at time 1 and time 2. SRS, Social Responsiveness Scale; ASD, Autism Spectrum Disorder; TD, typically developing children.

A mixed ANOVA revealed a significant Group × Time interaction (*p* < 0.001) on children’s SRS Total scores (**Figure 2**). When sex was included in the model, there were no sex effects or interactions, and therefore this factor was collapsed for further analyses. Furthermore, results showed a significant effect of time and group (*p* < 0.001). *Post hoc* analysis revealed a significant improvement of social functioning over time in children with ASD only (*p* < 0.001), but who were still impaired at both time points compared to TD children. Given adequate social functioning at baseline, the TD group showed very minimal change in social ability over 2 years (i.e., less than one standard score). Separate analyses (ANCOVA) were conducted controlling for the effect of age at baseline, to ensure that baseline age did not have an impact on the magnitude of change that occurred over time. This helped resolve concerns that potentially younger children have more “more room to grow” EF skills, compare to older children. When controlling for age, we found no significant Time × Age interaction on the SRS Total, indicating that effects of time on social function were not impacted by initial age of the participants.

Given minimal change of social skills over time observed in TD children, we examined the predictive link between change in scores of EF and social functioning over 2 years in children with ASD only. Preliminary Pearson’s correlations between change scores revealed a significant positive correlation between change in MCI and change in SRS Total (*r* = 0.45, *p* < 0.01) from T_1_ to T_2_. We found no significant correlation between change in BRI and change in SRS Total (*r* = 0.27, *p* = 0.11; see **Table 4**).

**Table 4:** Correlation matrix of change of EF and social functioning from T_1_ to T_2_ in ASD.

	1	2	3	4	5
(1) Change in SRS total	–				
(2) Change in BRIEF BRI	0.27	–			
(3) Change in BRIEF MCI	**0.45^∗∗^**	**0.56^∗∗∗^**	–		
(4) IQ	-0.11	-0.20	-0.15	–	
(5) Sex	-0.06	-0.08	-0.10	-0.01	–

*^∗^p < 0.05, ^∗∗^p < 0.01, ^∗∗∗^p < 0.001. Significant values after correction for multiple comparisons are in bold.*

Simple regression analysis in the ASD group indicated that improvements in BRIEF MCI significantly predicted improvement in SRS Total (*p* < 0.01) in ASD, accounting for 18% of adjusted variance (**Table 3**).

## Discussion

This is the first study to (1) track the development of everyday EF in children with and without ASD over 2 years utilizing parent ratings and (2) investigate how two domains of EF (behavioral regulation and metacognition) are associated with later social, emotional and behavioral outcomes. Results indicate that children with ASD showed persistent impairments over time in all aspects of everyday EF compared to TD. Furthermore, these early difficulties of EF were predictive of later social, emotional and behavioral problems in youth with ASD.

As expected, the current study showed that EF in TD children remained stable over time (i.e., this group is not expected to improve over time if they are performing at age-expected levels at baseline). Interestingly, children with ASD, who showed impaired EF compared to TD children at baseline, showed no improvements of BRI and MCI over 2 years, which is in line with previous cross-sectional studies investigating the development of EF as observed in everyday settings (i.e., informant-reported EF; Rosenthal et al., 2013; van den Bergh et al., 2014). Closer examination of EF subdomains revealed the greatest difficulties in flexibility (highest *T-* scores on the BRIEF Shift subscale) at both time points for children with ASD relative to TD, which is consistent with previous research (Russo et al., 2007; Granader et al., 2014; Wallace et al., 2016). While many studies employing laboratory tasks demonstrate that EF problems become less marked with age (Happé et al., 2006; Best and Miller, 2010; Pellicano, 2010), the present study documented significant EF difficulties in *everyday settings* as reported by parents that *persisted over time* in youth with ASD compared to TD children. Everyday EF deficits have been shown in adults with ASD as well (Wallace et al., 2016). These findings may suggest that while environmental demands increase dramatically as children enter adolescence (Blakemore and Mills, 2014; Brizio et al., 2015), refinements of EF problems are impaired in ASD, making it challenging for them to adapt to and keep pace with growing real-world demands.

The present study also found that prior estimates of everyday EF were predictive of key outcomes in ASD: emotional (i.e., anxiety/depression symptoms), behavioral (i.e., aggressive/oppositional behavior) and overall social functioning, consistent with the recent study by Gardiner and Iarocci (2018) of youth with ASD at a single time point. Specifically, behavioral regulation (i.e., BRIEF BRI) difficulties at baseline predicted internalizing symptoms (anxiety and depression) 2 years later in children with ASD. Furthermore, both behavioral regulation and metacognitive (i.e., BRIEF MCI) problems at baseline predicted externalizing symptoms 2 years later, specifically oppositionality, conduct and aggressive/disruptive behaviors. However, it should be noted that when controlling for BRI, MCI was no longer significantly associated with externalizing symptoms. Thus, these results indicate that deficits in EF related mainly to behaviors (i.e., inhibition, shifting, emotional control) that are most relevant for the development of future emotional and behavioral problems in ASD. Similarly, Lawson et al. (2015) reported that specific deficits in parent-reported cognitive flexibility predicted greater anxiety/depression and aggressive symptoms in childhood ASD. Moreover, parent-reported inflexibility in adults with ASD is associated with anxiety-related symptoms, while metacognition problems are related to depression symptoms, above and beyond the influence of attentional problems (Wallace et al., 2016). Our findings also parallel the results of studies employing lab-based tasks of EF, which have demonstrated links between poor shifting and inhibition (i.e., behavior regulation EF domains), but not working memory (i.e., metacognitive EF domain), and symptoms of anxiety in youth with ASD (Hollocks et al., 2014). A single longitudinal study by Andersen et al. (2015b) reported that behavior and emotional improvement over 2 years was *not* associated with increased verbal working memory capacity in ASD. One explanation for this discrepancy may be that EF related to behavior, which captures the ability to appropriately control behavioral and emotional responses, is more critical for emotional and behavioral functioning in this group than metacognitive processes of EF, such as working memory. Overall, the present study extends previous literature by demonstrating a 2 years predictive developmental association between everyday manifestations of EF and co-morbid emotional and behavioral psychopathology.

Analyses examining the association between change in EF and change in social functioning over time in ASD revealed that an improvement only in MCI (i.e., initiating, planning, organization, working memory, self-monitoring) predicted an improvement in social functioning across 2 years. Similarly, previous literature (non-longitudinal) has also linked real-world metacognitive deficits, but not behavior regulation, to weak adaptive social skills (Gilotty et al., 2002). That a reduction in social deficits is associated with improvement in MCI, but not BRI, suggests that the ability to take initiative, plan and carry out possible actions, maintain task-relevant information in mind, and monitor/control ongoing mental operations is particularly crucial for social development in ASD. Research has consistently reported an association between metacognitive skills and theory of mind abilities, which can be considered a proxy for social functioning (see Hughes and Leekam, 2004 for review). Additionally, interventions with a focus on metacognitive skills have demonstrated effectiveness in improving social functioning in children and adolescents with ASD (Kenworthy et al., 2014) and intellectual disabilities (Rosenthal-Malek and Yoshida, 1994).

The current study had a number of limitations. First, our sample was relatively small, and a larger sample may have yielded additional information regarding the relations between EF, emotional/behavioral functioning and social functioning, and would have allowed us to conduct more complex analyses. Furthermore, given our sample size, we were limited in the number of statistical analyses that could be conducted and, in turn, we focused on BRIEF index scores (i.e., BRI and MCI). Future research examining the longitudinal relations between EF sub-functions (i.e., BRIEF subscales), co-morbid psychopathology and social functioning is needed to capture more specific EF impairments that may be related to developmental outcomes in ASD. In the current study, we failed to find a relation between IQ and study variables; relations may have been discovered using separate measures of verbal and non-verbal IQ. We administered a 2-subtest version of the WASI and, consequently, were unable to examine verbal and non-verbal IQ scores in the present study. Additionally, we did not have CBCL measures at time 1 and were unable to explore whether change of EF over time was related to changes of behavior and emotional functioning. Lastly, findings are restricted to development over 2 years and further longitudinal investigations into adulthood are crucial to better understand the trajectory of EF, and its relations to social, emotional and behavioral functioning across the lifespan.

These findings represent the first steps toward characterizing the developmental trajectory of EF in everyday settings and its developmental links with important areas of functioning in ASD, and have a number of important implications. Findings suggest that the inclusion of EF assessment in standard mental health evaluations for children with ASD may allow for a better diagnosis and prognosis of social and emotional problems and will help inform treatment planning. Further, findings suggest that everyday EF deficits may be potential risk factors for the development of later social, emotional, and behavioral problems in children with ASD, and thus early interventions targeting EF abilities, alongside traditional psychosocial or autism therapies, could mitigate or possibly prevent poor social functioning or co-morbidity later in life. However, future studies investigating EF interventions are desperately needed to confirm these links and speculations. Existing studies of EF interventions in TD children have yielded improvement in not only EF (Diamond et al., 2007) but also externalizing and internalizing behavior (Riggs et al., 2006), although the long-term impacts of such interventions are not well defined. Recent investigations of an EF school-based curriculum (Unstuck and On Target!; Cannon et al., 2011) targeting cognitive flexibility and metacognitive skills adapted specifically for children with ASD demonstrated effectiveness in improving EF, classroom behavior and social skills (Kenworthy et al., 2014). Fisher and Happé (2005) also showed that EF training in children with ASD contributed to improvements in theory of mind, but not EF. Although this initial work is promising, it is limited and the impact of EF-based treatments on co-morbid psychopathology remains unknown; as such, more research is needed in this area. Furthermore, given that EF related to *behavior regulation* is more critical for later emotional and behavioral functioning, and EF related to *cognition* is more critical for social functioning, it may be beneficial to tailor treatment. For instance, specific interventions for inhibition, cognitive flexibility and emotional control (i.e., behavioral regulation) could yield potential improvements in anxious/depressive symptoms and a reduction in aggressive/oppositional behavior, whereas training in planning, initiation, working memory, self-monitoring and organization (i.e., metacognition) may result in improved social functioning. If early EF interventions are shown to improve social, emotional and behavioral outcomes in ASD, this would not only strengthen the evidence for links between these factors, but also provide important clinical insights. At this time, EF skills are not often the focus of interventions for children with ASD, and the implications of our findings support the possibility of adapting traditional treatment approaches to include EF training throughout childhood and adolescence. Doing so may enhance prevention of co-morbid psychopathology and promote social competence in youth with ASD, particularly during a time of cognitive development.

## Conclusion

In conclusion, our study found that prior estimates of everyday EF predict later social functioning and co-morbid psychopathology in youth with ASD. Specifically, findings show that EF related to behavior (i.e., behavioral regulation) are more critical for later emotional and behavioral functioning, whereas EF related to *cognition* (i.e., metacognition) are more critical for social skill development over time in youth with ASD. Findings support the importance of EF problems in influencing psychological and social outcomes in ASD, and as potential intervention targets, alongside traditional autism and mental health therapies. Future studies are needed to examine the effectiveness of EF-based interventions in diminishing co-morbid psychopathology and social difficulties in children and adolescents with ASD.

## Author Contributions

VV contributed substantially to conceptualization and study design, data acquisition, data analysis, interpretation of the data, drafting of the manuscript, and final approval of the manuscript. RL contributed to data acquisition, interpretation of the data, critically revising the manuscript, and final approval. KS contributed significantly to analyses and interpretation of data and editing. RM and MLS contributed to the interpretation of data, revising the manuscript for intellectual content, and final approval. MT contributed to conceptualization and study design, interpretation of the data, drafting of the manuscript, and final approval. All authors agree to be accountable for all aspects of the work in ensuring that questions related to the accuracy or integrity of any part of the work are appropriately investigated and resolved.

## Conflict of Interest Statement

The authors declare that the research was conducted in the absence of any commercial or financial relationships that could be construed as a potential conflict of interest.
