# Supplementary figures and images for: Longitudinal Examination of Everyday Executive Functioning in Children With ASD: Relations With Social, Emotional, and Behavioral Functioning Over Time
Source: Front Psychol. 2018 Oct 10;9:1774. doi: 10.3389/fpsyg.2018.01774 (PMC6191468; doi:10.3389/fpsyg.2018.01774)

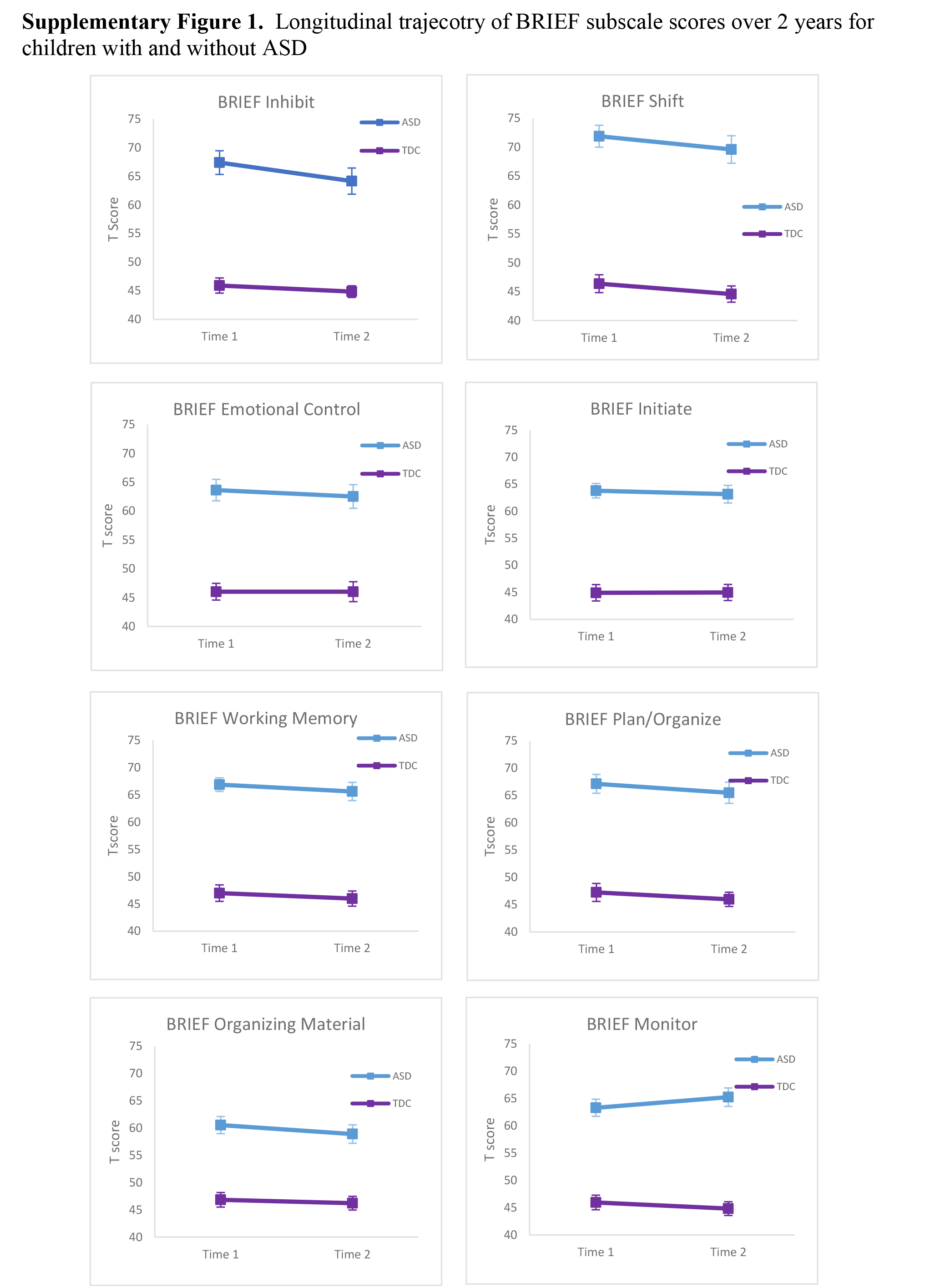

Supplement: Supplementary file 1 [file Image_1.TIF]
